# Salivary Biomarkers in Periodontitis: A Scoping Review

**DOI:** 10.7759/cureus.50207

**Published:** 2023-12-08

**Authors:** Sultan Abdulkareem Ali Alftaikhah, Rakhi Issrani, Muhsen Alnasser, Haifa Ali Almutairi, Osama Khattak, Azhar Iqbal, Namdeo Prabhu

**Affiliations:** 1 Dentistry, College of Dentistry, Jouf University, Sakaka, SAU; 2 Preventive Dentistry, College of Dentistry, Jouf University, Sakaka, SAU; 3 Research Analytics, Saveetha Dental College and Hospitals, Saveetha Institute of Medical and Technical Sciences, Saveetha University, Chennai, IND; 4 Restorative Dental Sciences, College of Dentistry, Jouf University, Sakaka, SAU; 5 Restorative Dental Sciences, College of Dentistry, Jouf University, Sakakah, SAU; 6 Oral and Maxillofacial Surgery & Diagnostic Sciences, College of Dentistry, Jouf University, Sakaka, SAU

**Keywords:** scoping review, salivary biomarker, periodontitis, prognostics, salivary, oral health, healthcare, diagnostics, biomarkers

## Abstract

Periodontal disease is a prevalent and potentially impactful oral health condition, ranging from gingivitis to severe periodontitis. Early detection and precise management are crucial in modern dentistry due to its prevalence and potential systemic health implications. Traditional clinical assessments and radiographic imaging have been the primary diagnostic tools. However, recent advances in oral diagnostics have introduced the concept of non-invasive, easily accessible salivary biomarkers. This review explores the evolving landscape of salivary biomarkers associated with periodontal disease, offering a comprehensive analysis of recent studies. It delves into the key findings, clinical significance, and potential impact of these biomarkers in revolutionizing periodontal disease diagnostics and treatment monitoring. The study emphasizes their diagnostic and prognostic capabilities, including their ability to assess disease severity, correlate with clinical parameters, aid in early detection, and enhance personalized treatment planning. As the field of oral diagnostics continues to advance, understanding the role of salivary biomarkers in periodontal disease management holds the promise of improving precision and effectiveness in oral healthcare. This review underscores the potential for salivary biomarkers to become integral components of routine periodontal care, offering a minimally invasive and patient-centered approach to oral health management.

## Introduction and background

Periodontal disease, a common yet often silent oral health condition, encompasses a spectrum of inflammatory disorders affecting the supporting structures of the teeth. Its progression, from the initial stages of gingivitis to more severe forms of periodontitis, can result in tissue destruction, tooth mobility, and even tooth loss if left untreated [1-3]. Given its widespread prevalence and potential impact on systemic health, the early detection, accurate diagnosis, and effective management of periodontal disease have become pivotal in modern dentistry [1].

Traditionally, the diagnosis of periodontal diseases has relied on clinical assessments such as probing depth, clinical attachment loss, and radiographic imaging [4]. While these methods remain fundamental, recent advances in the field of oral diagnostics have ushered in a new era where non-invasive, easily accessible biomarkers in saliva are gaining prominence [5, 6]. Salivary biomarkers have emerged as promising candidates for aiding in the timely identification, risk assessment, and monitoring of periodontal disease [7].

The primary objective of this review is to investigate and summarize the scientific literature pertaining to the identification of biomarkers in human saliva associated with periodontal diseases. Within this context, we will delve into the key findings and clinical significance of salivary biomarkers, as evidenced by a selection of representative studies. These studies shed light on the potential of salivary markers to revolutionize periodontal disease diagnostics and treatment monitoring.

In particular, we will examine the distinct biomarkers analyzed in these studies, their relationships with clinical parameters, and their diagnostic and prognostic capabilities. By synthesizing the collective knowledge from these investigations, this review aims to provide a comprehensive overview of the current state of salivary biomarkers in the context of periodontal health and disease.

As the world of oral diagnostics continues to evolve, understanding the role of salivary biomarkers in periodontal disease may not only enhance our ability to detect and manage this condition but also open new avenues for personalized, precise, and minimally invasive approaches to oral healthcare [8].

In the following sections, we will delve into the methodologies employed, key findings, and clinical implications of these studies to offer a holistic perspective on the potential of salivary biomarkers in the realm of periodontal disease management.

## Review

Methodology

Search Strategy

A comprehensive literature search was conducted using a systematic approach to identify relevant articles on the presence and role of biomarkers in saliva in periodontal disease. The search strategy utilized a combination of medical subject headings (MeSH) terms and keyword phrases. The key search terms included "biomarker," "salivary biomarkers," "oral fluid biomarkers," "saliva," "oral fluid," "salivary," "saliva markers," "periodontal diseases," and "periodontitis."

Database Selection

The search was conducted in PubMed, a comprehensive biomedical and dental literature source, by applying filters such as limiting the search to articles published in the last five years, articles in English, articles involving human subjects, and full-text articles.

Inclusion and Exclusion Criteria

The inclusion and exclusion criteria were applied to refine the selection. Articles on systemic diseases, obesity, pregnancy, oral cancer, autoimmune disorders, neurological disorders, and rheumatoid arthritis were excluded, as they could interfere with assessing salivary biomarkers for periodontal disease. After removing these articles, 62 remained. To ensure a more homogeneous population in the selected studies, articles that did not exclude or account for the impact of smoking were further excluded. Reviews were excluded to focus on primary research findings.

Screening

The initial search yielded a total of 195 articles based on the specified search criteria. After screening through the titles, irrelevant articles were excluded by two independent reviewers (MA and HAA). This initial screening reduced the number of articles to 81. After the removal of articles as per the exclusion criteria, only 39 articles were selected for abstract and full-text screening.

The abstracts of the remaining articles were reviewed by two independent reviewers (MA and HAA) to assess their relevance to the research objective. Articles that did not pertain to the identification or analysis of salivary biomarkers in periapical inflammation were excluded at this stage. Following the abstract screening, 16 articles remained. At any stage, if there was a conflict, a third reviewer (SAAA) made the final call.

Final Article Selection

The 16 selected articles underwent a comprehensive review of their full text. After a thorough evaluation, five articles that met the criteria for investigating salivary biomarkers in periodontal disease were finalized for inclusion in this review.

Data Extraction

For the selected articles, relevant data, including study design, sample size, identified biomarkers, key findings, and clinical significance, were extracted for incorporation into the review.

Data Synthesis and Review Composition

**Figure 1:**
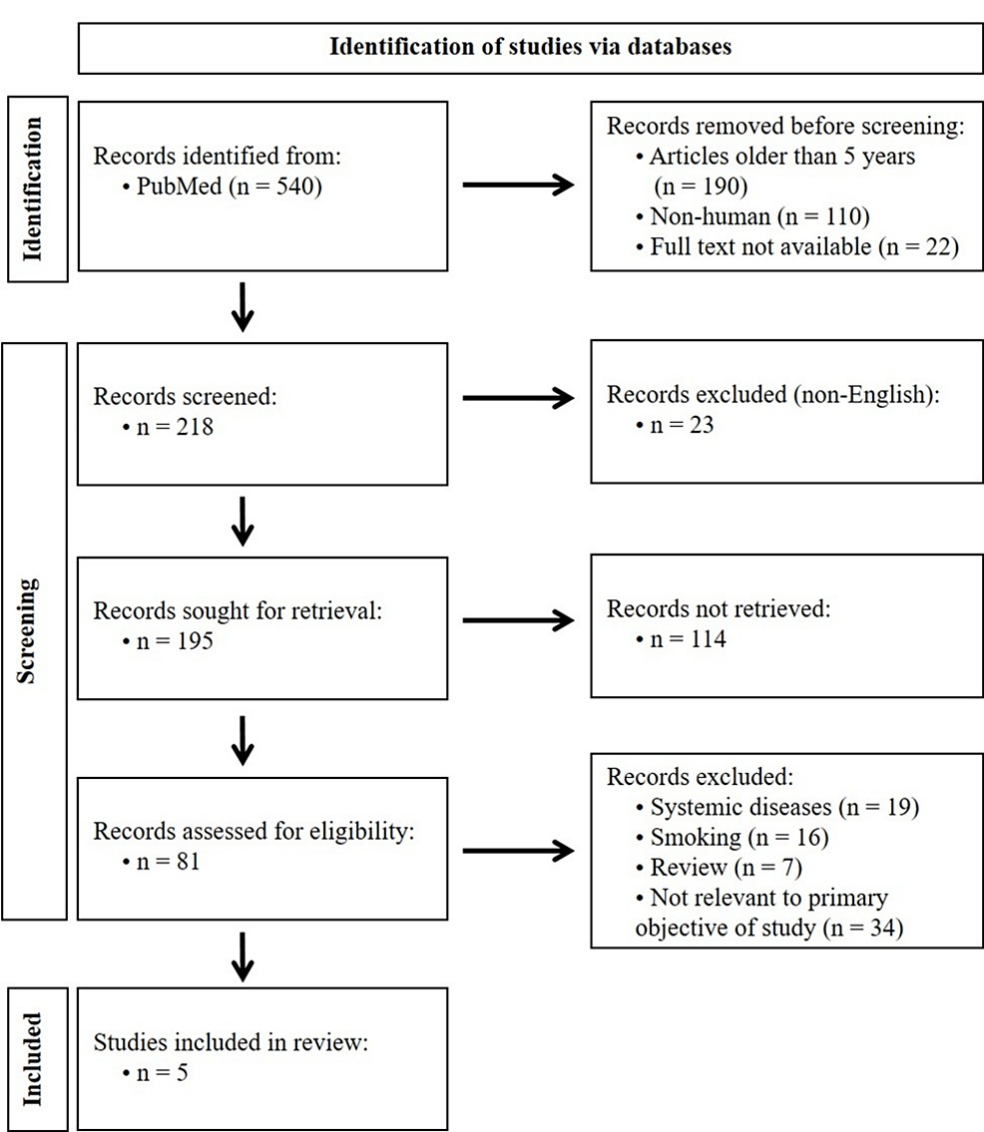
PRISMA flowchart of the search strategy PRISMA: Preferred Reporting Items for Systematic Reviews and Meta-Analyses

The findings from the selected articles were synthesized and are presented in the review article. A comprehensive analysis of the studies' methodologies, results, and implications for the diagnosis and management of periapical inflammation will be conducted. A flowchart of the search strategy is shown in Figure 1.

Results and discussion

The reviewed studies collectively enhance our comprehension of salivary biomarkers linked to periodontal disease. These biomarkers hold promise in terms of diagnostic and prognostic value, potentially revolutionizing clinical practice in dentistry. The first study highlights the association between reduced levels of CD9 and CD81 exosome-related tetraspanins in saliva and periodontitis, particularly in more severe cases. The tetraspanin proteins CD9 and CD81 play significant roles in saliva, particularly in the context of periodontal health. Reduced levels of CD9/CD81 exosomes in saliva have been associated with periodontal clinical status, indicating a potential link between these tetraspanins and the health of the periodontium. The continued significance of CD81, even after demographic adjustments, positions these exosomes as potential diagnostic or prognostic markers for periodontal disease, offering the potential for early detection and ongoing monitoring.

The second study uncovers increased levels of soluble urokinase plasminogen activator receptor (suPAR) and galectin-1 in the gingival crevicular fluid of individuals with periodontal disease, especially in chronic periodontitis patients. This discovery suggests their role as valuable diagnostic markers, potentially aiding in early disease detection and intervention.

In the third study, elevated salivary levels of matrix metalloproteinase 9 (MMP-9) and S100 calcium-binding protein A8 (S100A8) were observed in periodontitis patients and were correlated with clinical parameters. These findings propose the utility of salivary MMP-9 and S100A8 as diagnostic and prognostic indicators, reflecting disease severity and helping to identify individuals at risk or monitor disease progression.

The fourth study reveals significantly higher levels of salivary lactate dehydrogenase (LDH) and aspartate aminotransferase (AST) in periodontitis patients, positively correlated with clinical parameters. Salivary LDH and AST have the potential to serve as markers for assessing disease progression and severity, allowing clinicians to track disease development and tailor treatment strategies.

In the fifth study, significantly elevated levels of salivary cytokines, including interleukin (IL)-1β, IL-6, and tumor necrosis factor-alpha (TNF-α), were observed in periodontitis patients and strongly associated with clinical parameters. These salivary cytokines, particularly IL-1β, IL-6, and TNF-α, offer reliability in assessing the severity of periodontal disease, making them valuable markers for monitoring disease progression.

Collectively, these studies emphasize the potential of salivary biomarkers as non-invasive tools for the diagnosis and monitoring of periodontal disease. The diverse array of biomarkers investigated reflects the multifaceted nature of this condition. As research advances, these biomarkers may become integral in clinical dentistry, facilitating early detection, assessing disease severity, and enabling the development of more personalized treatment plans for individuals with periodontal disease. However, further research and validation studies are essential to confirm their clinical utility and standardize their use in dental practice. Table 1 summarizes the study design, sample, analyzed biomarkers, key findings, and clinical significance of the reviewed studies.

**Table 1:** Summary of the reviewed articles CD9 and CD81: members of the tetraspanin superfamily

Study title	Study design	Sample size	Analyzed biomarkers	Key findings	Clinical significance
Decreased salivary concentration of CD9 and CD81 exosome-related tetraspanins [9]	Cross-sectional study	149	CD9 and CD81 exosome-related tetraspanins in saliva	Lower salivary CD9 and CD81 exosome levels in periodontitis patients, especially with increased disease severity. CD81 remained significantly associated with periodontitis after adjusting for demographics.	Potential diagnostic or prognostic markers for periodontal disease.
Assessment of saliva and gingival crevicular fluid soluble urokinase plasminogen activator receptor (suPAR), galectin-1, and tumor necrotic factor-alpha (TNF-α) levels [10]	Cross-sectional study	60	suPAR, galectin-1, and TNF-α levels in saliva and gingival crevicular fluid (GCF)	Increased levels of suPAR and galectin-1 in the gingival crevicular fluid (GCF) of individuals with periodontal disease suggest their potential involvement in disease development. Notably, salivary suPAR levels were notably higher in chronic periodontitis patients.	Valuable diagnostic markers for periodontal disease, potentially aiding in early detection and intervention.
Diagnostic and prognostic ability of salivary matrix metalloproteinase 9 (MMP-9) and S100 calcium-binding protein A8 (S100A8) for periodontal disease [11]	Cross-sectional study	84	Salivary MMP-9 and S100A8 levels	Elevated salivary MMP-9 and S100A8 levels in periodontitis patients compared to controls. These markers correlated with clinical parameters such as probing depth and clinical attachment loss.	Salivary MMP-9 and S100A8 can serve as diagnostic and prognostic indicators, reflecting disease severity.
Association of salivary lactate dehydrogenase (LDH) and aspartate aminotransferase (AST) with periodontal diseases [12]	Cross-sectional study	130	Salivary LDH and AST levels	Salivary LDH and AST levels were significantly elevated in periodontitis patients. Both LDH and AST showed positive correlations with clinical parameters such as probing depth and clinical attachment loss.	Salivary LDH and AST are potential markers for assessing periodontal disease progression and severity.
Assessment of salivary inflammatory biomarkers and their association with periodontal disease severity [13]	Cross-sectional study	90	Cytokines (interleukin (IL)-1β, IL-6, and TNF-α) in saliva	Salivary IL-1β, IL-6, and TNF-α levels were significantly higher in periodontitis patients compared to healthy controls. These cytokines exhibited strong associations with clinical parameters.	Salivary cytokines, particularly IL-1β, IL-6, and TNF-α, can serve as reliable markers for periodontal disease severity and may aid in monitoring disease progression.

The growing interest in salivary biomarkers for periodontal disease marks a significant shift in dental diagnostics and treatment approaches [14]. The studies reviewed here emphasize the potential of these non-invasive markers for enhancing our understanding of periodontal health, diagnosing different disease stages, and predicting treatment outcomes. In this discussion, we delve into the implications of the findings from these studies.

Study Design and Sample Size

The diverse range of study designs, including cross-sectional and prospective clinical trials, used across these studies allows for a multifaceted exploration of salivary biomarkers in periodontal disease [15]. Cross-sectional studies, such as those by Tobon-Arroyave et al. [9] and Inönü et al. [12], offer valuable insights into biomarker associations with clinical parameters [16]. Conversely, prospective trials, exemplified by Kim et al., provide essential longitudinal data for understanding biomarker dynamics during treatment [17].

Sample size considerations, particularly in diagnostic and prognostic studies, are vital [18]. Tobon-Arroyave et al., Kim et al., and Zhang et al. address this by recruiting substantial participant cohorts, ensuring robust statistical power [9, 11, 13]. Adequate sample sizes enhance result generalizability and biomarker performance reliability [19]. However, a balance must be maintained between participant numbers and available resources.

Analyzed Biomarkers

The selection of biomarkers across these studies is diverse and comprehensive, reflecting the multifaceted nature of periodontal disease. The studies focus on various salivary proteins and DNA markers, including exosome-related tetraspanins (CD9 and CD81), suPAR, galectin-1, MMP-9, S100A8, and various cytokines (IL-1β, IL-17). This diversity mirrors the complex pathophysiology of periodontal disease, which encompasses inflammation, tissue remodeling, immune responses, and microbial interactions [20]. The integration of multiple biomarkers, as seen in Zhang et al.'s study, can enhance diagnostic accuracy by capturing various aspects of disease progression [13].

Key Findings

Several consistent key findings emerge across the studies.

Association with disease severity: Salivary biomarkers show promise in distinguishing healthy individuals from those with periodontal disease [21-23]. This distinction is based on substantial variations in specific biomarkers' levels in saliva samples from these groups.

Assessing disease severity is pivotal for clinical decision-making and guiding treatment planning. Salivary biomarkers, as shown by Kim et al., Lee et al., and Syndergaard et al., demonstrate potential as discriminative tools. Healthy individuals typically exhibit specific baseline saliva biomarker levels indicative of oral health balance. In contrast, individuals with periodontal disease display distinct biomarker concentration alterations, reflecting oral tissue inflammation [21-23].

These differences in salivary biomarker profiles provide valuable insights into an individual patient's disease severity. Quantifying and analyzing these biomarkers helps clinicians categorize patients into disease severity levels, from mild to severe. This stratification enhances accurate periodontal disease diagnosis and tailored treatment planning. In essence, salivary biomarkers' ability to differentiate healthy individuals from those with periodontal disease underscores their potential as objective, quantitative disease severity indicators. This information equips clinicians with data for precise patient-tailored treatment plans, enhancing periodontal disease management effectiveness [24]. However, further research and validation are needed to fully establish these biomarkers' clinical utility.

Correlation with clinical parameters: Biomarkers such as IL-1β, MMP-8, and S100A8 are extensively researched in periodontal disease. They are detectable in salivary samples and exhibit compelling correlations with key clinical indicators routinely used for periodontal health and disease assessment, such as probing depth and bleeding on probing [11]. In periodontal disease, increased probing depth indicates inflammation and tissue destruction. Interleukin-1β, MMP-8, and S100A8 have shown significant correlations with probing depth.

Literature shows that elevated IL-1β levels in the saliva are associated with deeper probing depths in periodontal disease patients. This suggests that as inflammation and tissue destruction within periodontal pockets intensify, salivary IL-1β concentrations increase [25]. This correlation's strength implies that salivary IL-1β levels reliably indicate pocket depth. The tissue-breaking enzyme MMP-8 has a positive correlation with probing depth. As periodontal disease progresses, leading to deeper pockets, MMP-8 levels tend to rise, reflecting ongoing periodontal tissue degradation [26]. The calcium-binding protein S100A8, which is connected to inflammation, also has a correlation with probing depth. Higher concentrations are often associated with deeper pockets, reinforcing its potential as a salivary biomarker for periodontal disease severity [27].

These correlations between salivary biomarkers and clinical indicators have critical clinical implications; they emphasize that salivary biomarkers reflect ongoing oral tissue inflammation and destruction. As disease severity increases, biomarker levels in saliva rise. Salivary biomarkers complement conventional clinical assessments, offering an objective, quantifiable dimension to periodontal disease diagnosis and monitoring and enhancing treatment planning precision. Using salivary biomarkers alongside traditional clinical parameters provides a comprehensive view of a patient's periodontal health, allowing tailored, patient-centered treatment [28].

Diagnostic potential: Zhang et al.'s study introduces an innovative approach by assessing combinations of salivary biomarkers, improving diagnostic accuracy, particularly in distinguishing gingivitis from periodontitis [13].

Combining multiple salivary biomarkers offers enhanced sensitivity and specificity. Gingivitis and periodontitis are distinct, with varying severity and inflammatory processes. Single biomarkers may not adequately capture this complexity [24]. Combining salivary biomarkers provides a more comprehensive view of underlying pathophysiological changes, improving diagnostic accuracy. The favorable area under the curve values in receiver operating characteristic analysis demonstrates their excellent diagnostic potential, effectively differentiating between gingivitis and periodontitis [13].

Early diagnosis of periodontal disease allows for more manageable, less destructive interventions. Combining salivary biomarkers, as demonstrated in the study, holds promise for identifying early periodontitis stages and optimizing patient care and outcomes [29]. Biomarker combinations also enable personalized treatment based on individual biomarker profiles, enhancing treatment efficacy and patient satisfaction. Tailored approaches can lead to more successful disease management.

Prognostic value: Periodontal disease management comprises various treatment modalities, from non-surgical procedures like scaling and root planning to surgery. Monitoring treatment responses is crucial. Kim et al.'s study highlights S100A8 and MMP-9 as biomarkers for assessing treatment response. These biomarkers are involved in periodontal disease-related inflammation, indicating ongoing inflammation and tissue damage [11].

Measuring S100A8 and MMP-9 levels before and after treatment provides insights into treatment effectiveness. A decrease post-treatment suggests reduced inflammation and a positive response. Periodontal disease is often chronic, requiring long-term management. Identifying patients at risk of progression or recurrence is essential. Prognostic indicators like S100A8 and MMP-9 play a role [30]. Persistent or elevated S100A8 and MMP-9 levels post treatment may indicate a higher recurrence or progression risk, prompting aggressive or personalized management strategies. Long-term monitoring aids in early disease resurgence detection, preventing further tissue damage and tooth loss.

Limitations

There are a few limitations associated with this review. Firstly, the number of investigations that we selected for our review can be deemed to be quite low, if compared to what an ideal review should look like, but the fact is that we were very stringent in our selection criteria for selecting studies and thus, only chose papers where the methodological quality was deemed to be fairly high. Moreover, most of the studies that we came across during our literature search were performed on laboratory animals, were animal-based, or were reviews, and as such, did not fit our objectives. Therefore, according to our observations, there is a probable need for clinical trials that examine the different types of salivary biomarkers in periodontal diseases. Secondly, only a few biomarkers were included in this review, although the list of salivary biomarkers associated with periodontitis is huge. The reason for including only a few biomarkers was that only the original papers from the last five years were included in this review. Thirdly, most of the studies included in this study aid in the diagnosis of the severity and progression of periodontitis but have limitations in detecting disease activity at each individual tooth site. Finally, the fact that the concentration of biomarkers can be affected by the saliva flow rate, circadian rhythm, age, the physiological status of the patients, and other factors raises concern over the accuracy and reproducibility of diagnoses using salivary biomarkers.

Clinical significance

These findings have significant clinical implications for periodontal disease management. Salivary biomarkers offer non-invasive tools for early disease detection, risk assessment, and treatment monitoring. Their diagnostic and prognostic potential may lead to personalized treatment strategies, optimizing patient care and outcomes. Integrating salivary biomarkers into routine periodontal assessments could enhance clinical decision-making precision and efficiency. Dentists may soon have access to a comprehensive biomarker panel combined with traditional clinical evaluations, providing a holistic view of periodontal health.

Future directions

Although recent studies have highlighted the potential of salivary biomarkers for diagnosing periodontal diseases, further research and clinical applications are necessary to fully explore their capabilities. While these biomarkers have shown promise in providing valuable information to dental professionals for more accurate diagnosis and treatment planning, there are still several challenges that need to be addressed. Currently, the tests used for periodontal disease diagnosis based on salivary biomarkers have limitations such as a small number of potential biomarkers, a lack of real-time assessment, and reliance on general microbial and inflammatory cytokines that may not be disease-specific.

To make salivary diagnostics of periodontal diseases clinically relevant, there is a need for better bioinformatics tools to discover more validated biomarkers with disease discriminatory power. The test also needs to be performed in real-time, allowing for immediate evaluation of the patient's periodontal status while they are in the dental office. Finally, biomarkers should not only be able to diagnose the disease but also predict the risk of future disease activity using simple and affordable means.

## Conclusions

The present review has explored the evolving landscape of salivary biomarkers in the context of periodontal disease. Through a systematic analysis of recent studies, we have highlighted the diagnostic and prognostic potential of various salivary markers, including CD9, CD81, suPAR, galectin-1, MMP-9, S100A8, LDH, AST, and pro-inflammatory cytokines (IL-1β, IL-6, and TNF-α). These biomarkers offer valuable insights into periodontal disease, from early detection to severity assessment and treatment planning. Their non-invasive nature and ease of sample collection make them attractive tools for clinicians and researchers alike. As we look toward the future, further research, discovery of disease-specific salivary biomarkers, standardization of assays, and integration into clinical practice will be pivotal in realizing the full potential of salivary biomarkers in oral healthcare. Additionally, accurate and real-time assessments of periodontal diseases for the general public, either at home or at the dental office, are much needed. The journey to personalized, precise, and patient-centered periodontal disease management continues, with salivary biomarkers paving the way for more effective and efficient approaches to oral health. Although challenges remain ahead, using saliva to gauge periodontal health appears bright for future applications to aid in the diagnosis of periodontal diseases and the prediction of periodontal treatment outcomes.
